# Deep Learning-Based Medical Images Segmentation of Musculoskeletal Anatomical Structures: A Survey of Bottlenecks and Strategies

**DOI:** 10.3390/bioengineering10020137

**Published:** 2023-01-19

**Authors:** Lorenza Bonaldi, Andrea Pretto, Carmelo Pirri, Francesca Uccheddu, Chiara Giulia Fontanella, Carla Stecco

**Affiliations:** 1 Department of Civil, Environmental and Architectural Engineering, University of Padova, Via F. Marzolo 9, 35131 Padova, Italy; 2 Department of Industrial Engineering, University of Padova, Via Venezia 1, 35121 Padova, Italy; 3 Department of Neuroscience, University of Padova, Via A. Gabelli 65, 35121 Padova, Italy; 4 Centre for Mechanics of Biological Materials (CMBM), University of Padova, Via F. Marzolo 9, 35131 Padova, Italy

**Keywords:** medical imaging, ultrasonography, MRI, X-ray, CT, musculoskeletal system, anatomical structures, segmentation, artificial intelligence, deep learning

## Abstract

By leveraging the recent development of artificial intelligence algorithms, several medical sectors have benefited from using automatic segmentation tools from bioimaging to segment anatomical structures. Segmentation of the musculoskeletal system is key for studying alterations in anatomical tissue and supporting medical interventions. The clinical use of such tools requires an understanding of the proper method for interpreting data and evaluating their performance. The current systematic review aims to present the common bottlenecks for musculoskeletal structures analysis (e.g., small sample size, data inhomogeneity) and the related strategies utilized by different authors. A search was performed using the PUBMED database with the following keywords: deep learning, musculoskeletal system, segmentation. A total of 140 articles published up until February 2022 were obtained and analyzed according to the PRISMA framework in terms of anatomical structures, bioimaging techniques, pre/post-processing operations, training/validation/testing subset creation, network architecture, loss functions, performance indicators and so on. Several common trends emerged from this survey; however, the different methods need to be compared and discussed based on each specific case study (anatomical region, medical imaging acquisition setting, study population, etc.). These findings can be used to guide clinicians (as end users) to better understand the potential benefits and limitations of these tools.

## 1. Introduction

In recent years, several deep learning tools have been implemented for the segmentation of anatomical structures to support a wide range of clinical applications for rapid and high-precision evaluation. Deep learning tools for the segmentation of the musculoskeletal system have been proposed to assist clinicians with applications in the field of computer-assisted surgery [1,2]; the facilitation of clinical decision making for treatment planning [3,4]; identification of biomarkers [5]; tissue landmarking [6]; model reconstruction [7,8,9], for example, non-invasive (in silico) simulation or virtual reality [10]; analysis and quantification of structure properties (i.e., shape, area, volume, thickness, and other texture features) [11,12]; body composition analysis [13,14,15] and bone assessment [11,16,17] for the prognosis of health conditions in terms of risk profiling and stratification; and so on.

Image segmentation is a process used to simplify the representation and analysis of an image and it consists of the classification of pixels to localize and delineate the shape of the objects represented [4,18]. The segmentation output highlights the anatomical region of interest (ROI), i.e., a tissue, to increase the precision of a medical intervention. However, the accuracy of manual segmentation depends on the skill and experience of the operator, leading to significant intra- and inter-observer variability and time consumption, which are critical bottlenecks in the workflow. This indicates the need for the creation of automated solutions [4,7]. In this regard, artificial intelligence (AI) approaches, such as deep learning (DL) have become increasingly popular methods for solving various automated computer vision tasks, such as the abovementioned segmentation. The advantage of DL algorithms is their ability to “learn” complex relationships from large datasets in a self-taught way, with minimal operator-imposed assumptions and without explicit knowledge of the data in terms of features to identify objects [4,19,20,21]. Prior to the recent advances in DL solutions, intensity-based approaches were a common choice; however, they have limitations due to the strong influence of imaging artifacts and variations in the intensity of different organs, which leads to inconsistent and misleading interpretations of the results [22]. To overcome these limitations, the deep learning paradigm with neural networks has been successfully proposed.

The term neural network is derived from neuroscience and these algorithms mimic the brain mechanism, that is, the brain is a gigantic network of neurons and the neural network is a network of nodes [23]. A variety of neural networks can be achieved by combining different node connections to learn the different features of an object and solve different tasks. Computationally speaking, a node behaves in relation to a mathematical operator (also known as an “activation function”) that receives input signals from the outside and multiplies them by weight values that represent the associations of neurons (synapses) and are updated during the training process. Multilayer neural networks are called deep neural networks [23]. Each layer can extract specific information from an initial input (i.e., a bioimage). Subsequent layers of the network combine the information of the previous layers, thus network architectures learn to detect features such as color and edges in their early layers, while in deeper layers, networks learn to detect more complicated features (with a more semantic meaning). In this way, the network learns from the data which significant features to identify and segments the anatomical structures.

The use of deep learning segmentation algorithms for medical applications needs to be discussed based on the computational strategies implemented for their development. In this way, the clinician, as an end user, does not obtain false interpretations of the results. For this reason, the aim of this investigation was to analyze the current state-of-the-art solutions for segmentation, with a focus on musculoskeletal structures, using deep learning approaches. The novelty of this work is to provide clinical experts with an overview of the challenges that have been faced in previous studies in the literature and the related solutions that have been implemented to develop an automated artificial intelligence tool that is capable of investigating (segmenting) musculoskeletal structures from bioimages and designed to meet today’s clinical needs. These results contribute to our understanding of the limits and advantages of the use of such tools in clinical practice.

## 2. Materials and Methods

The results of this systematic review were reported according to the Preferred Reporting Items for Systematic Reviews and Meta-Analyses (PRISMA) [24]. A search was performed in the PUBMED database that included articles published up until February 2022. The following keywords were included: deep learning, musculoskeletal system, segmentation (title). In total, 140 articles were obtained as the query output and they were reviewed with an initial selection of the titles and abstracts, against the inclusion/exclusion criteria by two reviewers, L.B. and A.P. Works concerning animal studies, brain/nerves/heart/gastric apparatus/eyes (globes, orbital fat)/osteoclasts/myofibers/skin segmentation, scanned-film mammograms/staining imaging, not in English, not found available online in full text, not concerning anatomical structures, and where segmentation was not the main topic (e.g., publicly available database articles) were excluded. A total of 112 relevant articles were fully or partially (if articles include only a partial discussion on the current topic) revised by the same reviewers. Of these, a total of 101 articles were considered eligible for the current dissertation (these are reported in the tables below and the approximate percentages, in accordance with the exclusion criteria). Another 14 documents (articles/books) were added to support the general discussion (not reported in the tables below and the percentages, not in accordance with the exclusion criteria). The PICO framework was used to guide the evaluation of the studies. Four other independent and expert reviewers (two engineers, F.U., C.G.F. and two clinicians, C.S., C.P.) supplemented this systematic review with additional evaluations.

## 3. Results

In general, deep learning models are trained/validated/tested using two elements: a set of labeled data (annotated images recognized as ground truth/gold standard) and a neural network architecture that contain many layers [21].

Starting with the pipeline of a generic deep learning tool for the segmentation of musculoskeletal structures from medical images, the purpose of this section is to present the main challenges and the related solutions that have been implemented in the literature for tool development.

### 3.1. Musculoskeletal Structures and Medical Imaging

The literature shows that deep learning algorithms have been developed for different imaging modalities for the segmentation of a variety of musculoskeletal structures. In terms of anatomical structures, we found solutions for the following: 33% were related to the lower limbs (bones, muscles, joint, knee cartilage/meniscus/ligament), 8% to the upper limbs (bones, shoulder muscles, tendons), 34% to the trunk (vertebrae, disc, muscles, ribs), 3% pelvis (bones, muscles), 14% to the head (bony orbit, mandible, maxilla, temporal bone, skull), and 8% to the whole body (bones, muscles). Concerning medical imaging, 39% of the studies used magnetic resonance imaging (MRI), 9% used ultrasonography (US), 41% used computed tomography (CT), 9% used X-ray, and 2% used pluri-imaging modalities (see Table 1).

In relation to the imaging modality, the main difficulties are attributed to factors such as variable image contrast, the inherent heterogeneity of the image intensity, image artifacts due to motion, spatial resolution (e.g., in low-resolution images, the cervical vertebrae become a single, connected spinal region which is misleading to the investigation) [26,87]. Therefore, some authors [2,18,62] have integrated multimodal imaging information to combine the benefits of individual modalities.

### 3.2. The Challenges of Database Construction

Bioimaging “mining” for clinical purposes could be extremely powerful under certain circumstances that rely on raw data acquisition, selection and processing, to solve bottlenecks such as a small sample size and data inhomogeneity.

#### 3.2.1. Strategies for a Small Sample Size

The success of any deep learning approach is highly dependent on the availability of a quality dataset (of labeled images) to train the network by using many samples (“big data”) [100]. Then, once the network has been successfully trained/validated/tested, it can be used directly on new images acquired during the clinical routine. To build the ground truth (mask images), the reference data are manually or semi-automatically segmented, coarsely or finely. This process usually involves laborious manual labeling, where annotations are performed by experienced observers. Using multiple annotators may offer a more reliable representation of multicenter studies (where different guidelines may be present). However, as reported by Brown et al. 2020 [106], it is also likely that manual segmentation is imperfect, which consequently affects the accuracy of the network performance. Therefore, a proper number of images should be acquired to ensure intra- and inter-operator reliability. Although we frequently found there was a scarcity of labeled training data, it is difficult to obtain a large and publicly available dataset due to the security of patient information, data privacy, lack of data-sharing practices between institutions, and so on [100].

The risk in training a network with a small set of data is that it can degenerate into an overfitting problem, when a significant gap between training and testing error occurs and the network is extremely sensitive to small changes in data representation.

**Figure 1:**
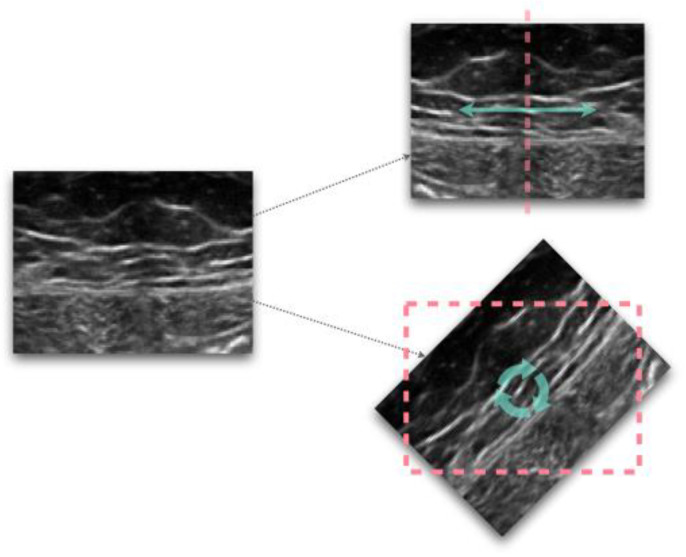
Examples of image transformations, flipping (top) and rotation (bottom).

Data augmentation, which consists of artificially increasing the number of training samples, is commonly implemented to solve the problem of overfitting and to boot network efficiency. The choice of its implementation is very context-specific. In this survey, data augmentation was performed by approximately half of the authors, and most of the time this involved applying various image processing techniques (i.e., more than 90% of them applied affine transformations) to the dataset (see Figure 1). Other recent techniques for data augmentation include various generative approaches. Among them, the generative adversarial network (GAN) is a deep learning method that identifies the intrinsic distribution of a dataset and exploits it to generate realistic synthetic samples [100]. Nikan et al. 2021 [10] reported that in case of samples with a low resolution, image artifacts or variations due to acquisition with different scanners, the data augmentation process also has the advantage of increasing the robustness of the model itself.

To manage small sample datasets, another solution is the transfer learning technique, which was implemented in more than 10% of the analyzed papers. The aim of transfer learning is to make a pre-existing algorithm reusable for a new dataset since it consists of using a pre-trained network (trained with another contextual dataset), and customizing it to the specific segmentation task so that the algorithm will only learn specific features for the new data (e.g., new data coming from new centers) [21] (see Table 2).

#### 3.2.2. Image Pre-Processing Techniques for Uniform Data Distribution

One of the biggest problems related to building the database is that the data often comes from different hospitals, it has been acquired with different devices, it has different resolutions/noises/illumination, and lacks repeatability; thus, the data includes large variations among different subjects/the same subjects monitored at different times, and therefore the dataset must be pre-processed [17,108]. In general, for optimal computer visual outcomes, attention to image pre-processing is required so that the image features can be improved by eliminating unwanted falsification [109]. So as to not alter the informative content of the images, it is essential to know the properties and the potential variability of the anatomical structure to be segmented, the studied population, the questions being investigated, and the robustness of the subsequent processing and analysis steps. For instance, in the case of image intensity normalization, the tissue taken as a consistent reference should always be present in the image and unlikely to be affected by pathological processes [106].

**Figure 2:**
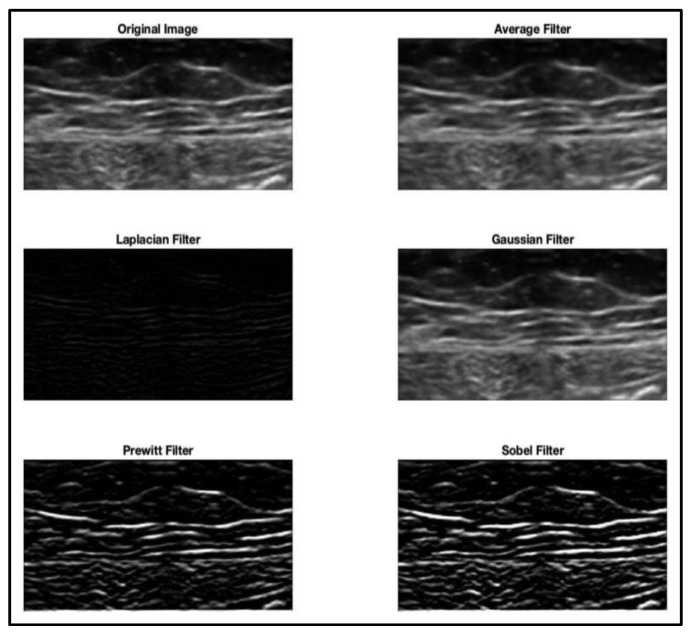
Image pre-processing: filtering techniques applied to the native image.

More than half of the authors (57%) implemented a pre-processing phase before the deep learning training step. The most commonly implemented pre-processing techniques were normalization; histogram equalization, which is used to increase the contrast of the image by spreading its intensity values; and intensity-based/dimensional-based filtering (see Figure 2).

Meanwhile, cropping and resizing methods (downsizing) were generally used to save memory space and are fundamental to match the dimensional requirements of the network input. When the region of interest (foreground) is small in relation to the background, then operations (such as resampling, downgrade in resolution, cropping) do not preserve the informative content of the tissue, thus leading to the loss of object details and surrounding relative contextual data. Instead, a two-pass run is a potential solution, where the native image should be processed in the first pass to allow the network to learn other significant determinants that may not be detected in the pre-processed one [14] (see Table 3).

As reported by Klein et al. in 2019 [103], sometimes it is necessary to perform expensive pre-processing and post-processing phases. For example, computer-aided diagnosis systems (which are rapidly developing with the help of modern computer-based methods and new medical-imaging modalities) generally require image pre-processing for image enhancement [110]. When there is no need for complex processing refinement, as highlighted by Norman et al. [5], there is a noteworthy improvement in performance time.

#### 3.2.3. Training/Validation/Testing Subsets Assignment

There are several strategies that are applied for the splitting of training/validation/testing subsets from the ground truth collection. In general, a training set is used for model training and construction, a validation set is used to monitor the model training process and observe the training effects, a test set is used to assess the generalization capabilities of the model [17]. In the case where the network is tested on a group of images that are greatly different from those used for training, the performance may decrease significantly. Generally, training/validation/testing subset assignment is performed by proportional splitting (e.g., 70%: 15%: 15%).

As reported by Goodfellow et al. 2016 [111], one procedure that uses all the examples in the estimation of the mean test error is k-fold cross validation, which was adopted by more than 20% of the authors in this survey. With this method, k-times a dataset is split into k equally sized subsets (one used for validation, the others are used for training). This process is then repeated k times. Then, the performance must be evaluated on a different test set. According to Rampun et al. 2019 [112], in some cases it is not necessary to perform a cross-validation, for example, when the number of training images is sufficient because cross-validation is extremely time consuming.

A bottleneck that should be considered in both of the abovementioned methods, is the redundancy in the data content. Redundant information can occur, for example, in the case of patients with multiple scans or images where the background is more prevalent compared to the foreground [10]. The latter issue can be solved through patch-based techniques, but as argued by Chen et al. 2019 [50], patch-based deep learning approaches have two main drawbacks. First, the receptive field of the network (region of an image in which the network is “sensitive” to features extraction) to the chosen patch-size is limited, and may only consider the local context. Second, the time required to run complex patch-based methods makes the approach infeasible when the size and number of patches are large.

In addition to the above considerations, to acquire representative subsets, Ackermans et al. 2021 [69] suggested the importance of discarding potential outliers. On the other hand, as noted by Liebl et al. 2021 [76], it is also essential that the database be as complete as possible with anatomical variants to allow for the development of robust and accurate segmentation algorithms.

### 3.3. Neural Network Architectures Applied to Musculoskeletal Structures Segmentation

The segmentation of anatomical structures can be performed based on the availability of bioimaging datasets through 2D or 3D approaches; however, combining the benefits of both solutions remains a challenge. The main reason the 2D method is popular is that it does not require an oversized dataset (using each slice as a network input, thus increasing the number of images, and consequently improving the performance and generalization of the network) and it is also very economical in terms of its computational and memory requirements. However, a 2D method does not fully utilize all sequence information (for example, between slices). This limitation could be overcome by using a 3D network capable of improving the continuity of the sequential slices and better segmentation of the small parts of the organs [4,113].

As trade-off strategies, the models could be trained by alternating the input batches by using batches of different planes (axial, sagittal, coronal) in such a way that the network learns to segment structures independent of the viewing direction, as proposed by Klein et al. 2019 [103]. Alternatively, a mixed two-steps algorithm could be implemented: a 2D organ volume localization network, followed by a 3D segmentation network, as discussed by Balagopal et al. 2018 [4].

In fact, over the years, various network architectures have been developed to segment musculoskeletal structures. One of the most popular models of the convolutional neural network (CNN), a type of artificial neural network that is widely used in the imaging domain, is the U-Net graph in its original form as proposed by Ronneberger et al. 2015 [114] or a modified version, which is utilized to solve 2D or 3D tasks. In this survey, the U-Net-based network was chosen by more than 60% of the authors (see Table 4). Indeed, as reported by Zhou et al. 2020 [11], the U-Net deep learning architecture has been proven to be effective in biomedical image segmentation tasks, even with limited data availability.

However, the choice of network architecture is driven by the case under study, which could guide the implementation of a new ad hoc design with a customized structure, as chosen by Kuang et al. 2020 [61].

To further improve the segmentation capability of the network, one design architecture strategy that could be considered is the introduction of an attention module, as proposed in [31,39]. Attention mechanisms can support the model to invest more resources in important areas of the structures present in an image, thus focusing on regions instead of analyzing the entire field of view. For models with moderate network depth, adding an attention module can improve the performance.

### 3.4. Network Training/Validation/Testing Process

Once the dataset and the network architecture have been defined, then the network should be trained/validated and tested to evaluate its performance through the use of different score indices.

**Figure 3:**
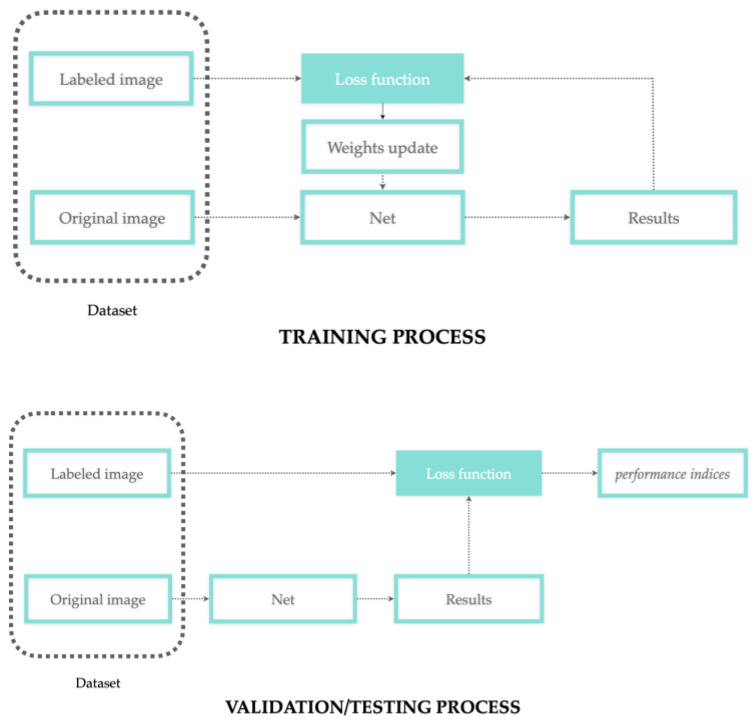
Training process with weights update workflow (top); validation/testing process workflow performed on different subsets (bottom).

In supervised learning, network training is the process where the segmented images, as the net output, are compared with a group of labeled images, as the net input, until the convergence between input and output is obtained (as error reduction). In other words, supervision means that each input image is coupled with a label, and this association is learned from the network to predict a specific output. During the learning process, the network weights are updated to reduce the error between the experts’ annotation (ground truth/labeled images) and the network prediction. This error is then evaluated and quantified through validation and testing processes. According to the concepts discussed in the previous sections regarding data labeling and weights update, Figure 3 reports a more detailed representation of the training/validation/testing process.

To reduce the burden of manual pixel-level annotations, so-called “weak” supervision has also attracted significant interest since, as explained by Kervadec et al. 2019 [115], it consists of annotating images in the form of partial or uncertain labels (e.g., bounding boxes, points, scribbles, or image tags).

#### 3.4.1. The Network Learning Process

During the training process, the difference between the expected output and the predicted one is estimated by the loss function. The training loss is used to update the weights and biases, while the validation loss determines whether the learning rate parameter (which regulates the way the network learns the problem, where a higher learning rate means faster but also sub-optimal training) is lowered or the training is stopped [99]. In fact, if the validation loss plateaus, according to different authors, the model training can be terminated [19] or the learning rate decays [4].

The most common loss functions used by the papers reported in this survey were the DICE function or variants (27%), cross entropy or variants (23%), or a combination of both (7%), see Table 5. Weighted terms in the loss function are a strategy that is utilized to solve class imbalance problems, and to make sure that different objects equally contribute to its quantification.

#### 3.4.2. The Network Performance

Comparing different deep learning architectures is a difficult task, and a network performance that is comparable to human observers could be considered a strong indicator of practical clinical utility [116]. However, different authors have chosen disparate performance indicators to evaluate the results according to each specific case. For example, as reported by Ackermans et al. 2021 [69], for a clinically useful algorithm that identifies patients at high risk for sarcopenia, the number of false negatives should be as low as possible as this represents a harmful health condition if not treated. Conversely, we may be willing to tolerate slightly more false positives, as sarcopenia treatment involves better nutrition and more functional activity, which are unlikely to harm anyone.

Thus, to validate the process, it is important to compare the selected technique with manual segmentation and with other network architectures. The most used indices to quantify network performance in terms of the overlap between ground truth and network predictions were the DICE index (DSC) (85%), Intersection over Union (IoU or Jaccard Index) (30%), Hausdorff distance (HD) (18%), and surface distance (SD) (18%). The HD and SD indices are generally used in case of 3D model reconstruction from multiple slices segmentation (see Table 6).

Another way to quantify the network performance is the run time of the predictions since it is crucial for the clinical real-time applicability of the tool. As evaluated by Ackermans et al. 2021 [69], the segmentation timing may also vary greatly in relation to different hardware.

An additional drawback in terms of timing efforts, is the tuning of the network hyperparameters (parameters set by users to control the learning process), which is performed for model optimization (such as the previously mentioned learning rate). This operation can be done based on a grid search or random search process [18], e.g., by trial and error [10,68,69]. Bayesian optimization is another method that is computationally less expensive than a grid search and that often converges to an optimal solution more rapidly than a random search [18,19].

Nonetheless, some authors have highlighted that network convergence could also be influenced by factors not strictly related to software or hardware development, such as the clinical conditions of patients under evaluation. For example, as demonstrated by Hemke et al. 2020 [13], a variation in accuracy can occur when a network trained with images from overweight subjects is applied to images from subjects with a low body mass index (BMI).

#### 3.4.3. Post-Processing Operations

After the abovementioned steps, different post-processing strategies could be implemented to ensure a more accurate outcome, to improve consistency, to refine the predictions, and correct mislabeling errors. Different post-processing techniques were selected by 18% of the surveyed authors for segmentation refinement (with morphological operations such as erosion/dilation or with dimensional thresholding) (see Table 7).

In any case, after the segmentation process, it is crucial to restore the properties of the original image, both in terms of resizing and resolution, as neural networks generally work with square matrices.

## 4. Discussion

Starting with the pipeline of a generic deep learning tool for the segmentation of musculoskeletal structures from medical images, the purpose of this systematic review was to offer a quick overview to clinical experts of the solutions proposed in the literature to fulfill the segmentation task, and to better clarify the potentiality and limitations in the usage of similar tools to complement their daily practices.

The results demonstrated that several solutions have been proposed in recent years for the segmentation of musculoskeletal structures for a variety of body parts (lower limbs, trunk, upper limbs, head, pelvis) with different bioimaging modalities (CT, MRI, X-ray, US) for clinical application, where automation is key to reduce the human effort involved in manual annotations (which are time consuming and prone to errors). Such tools should perform reliable and fast object characterization through a user-friendly interface, reduce costs and contribute to large-scale clinical process management and scale-up.

Tool feasibility relies, on a case-by-case basis, on its application, the anatomical structure to be analyzed and the bioimaging technique used for the investigation. The training/validation/testing of segmentation algorithms on non-public and single-institution datasets makes the comparison with published results infeasible due to the different conditions—in terms of the quantity of test images, the imaging parameters and hardware, the pathological status (or none) of the patients, and so on [116]. Therefore, the network should be trained/validated/tested with different inputs (in terms of the scanner setup/manufacturer, patient group, etc.) with the aim of simulating the variability of clinical practice. Moreover, to reduce redundancy, uniform data distribution and avoid biases that could lead to the misinterpretation of net outcomes, the database construction pipeline needs to include some fundamental operations such as the pre-processing step (which was implemented by >50% of the surveyed authors) and discarding the outliers. However, these operations must be performed without altering the information content in the image. In light of the above considerations, it is crucial to acquire a large amount of reliable data, or where not possible, to implement solutions such as data augmentation (which was chosen by 50% of the authors in the current survey and implemented by >90% of them through affine transformations) to enlarge the sample size and avoid problems such as overfitting.

The comparison between different methods has to be discussed on the basis of each specific case study (anatomical region, medical imaging acquisition setting, study population and so on); however, some common trends emerged from this investigation. These included the network architecture (>60% of authors chose U-net, in its original or modified version), loss function (27% chose the DICE function or variants, 23% chose cross entropy or variants, 7% chose a combination of both), post-processing refinement (18% of the authors used morphological operations or dimensional thresholding) and outcome indicators (85% DSC, 30% IoU, 18% HD, 18% SD) to evaluate the goodness of predictions.

Bottlenecks such as small sample size, data inhomogeneity and imprecise segmentation could be solved by data augmentation and pre- and post-processing operations, respectively, but the impact of such computational solutions should be considered in regard to the results to avoid false interpretations. Moreover, the accuracy of the predictions of an algorithm is a meaningful quantity not only when obtaining high values but also when the network learning process has been properly designed, with training as exhaustive as possible in regard to the variance of real cases, as encountered in daily practice.

Deep learning is a widely used solution due to its ability to learn the useful representation of an object from a bioimage in a self-learned way and without prior super-imposition of user-designed features (thus exceeding the limitations of traditional machine learning methods). For instance, this is the reason why vertebra segmentation relies on the integrity of intervertebral discs and is limited in the case of disc disease. In other words, a thoracic vertebra is identified not only due to its intrinsic features, but also due to the fact that it lies close to a disc (the disc is an extrinsic feature for vertebra that becomes a “landmark for the network” in this specific case of backbone segmentation). This also explains the limitation incurred when using patch-based approaches, where only limited contextual information is extracted and concur with outcomes predictions. Thus, the deep learning paradigm allows us to simultaneously investigate multiple pieces of information from an input, and to understand the way they are integrated and mutually influenced by each other. Therefore, network learning from huge amounts of data might suggest new biomarkers as predictors of musculoskeletal diseases through bioimaging analysis (potentially overcoming the limitations of human perception).

A “deep” knowledge of the workflow for tool development could support the evaluation of current or new software solutions for clinical applications, where the paradigm of the “patient at the center” requires tailored analysis and optimized settings. A segmentation tool could potentially increase the effectiveness of a physical therapy (i.e., laser, radial shock waves, etc.) if it could perform real-time parameter tuning, precisely based on each specific scenario and the conditions of the musculoskeletal structure, thus focusing the intervention on the precise level of the altered structure itself. In practice, a similar deep learning segmentation tool could help to guide clinicians in a customized investigation of the structures potentially implicated in a specific condition, in planning the best intervention, in re-evaluating treatments’ effectiveness and in monitoring their follow up.

The purpose of the current survey was to provide clinicians with a quick overview of the milestones for developing deep learning tools and to understand their applicability.

The challenge of this type of research was to overcome the problem of the different nomenclatures used by different authors to define the same topic, to identify common strategic solutions (e.g., network architectures, performance indicators and so on) and report the relative percentages (if significant).

The limitations of this type of research include the focus on the analysis on the musculoskeletal system and on the segmentation tasks. Further research could be conducted on other systems and tasks such as classification.

The search could also be extended to other topics such as network optimization (linking the performance of the techniques with the overall training and error optimization strategies) and publicly available databases for network training/validation/testing.

Future work could also include numerical statistics (in terms of performance indicators resulting from different tools) and the definition of a rating scale that compares different items (for example, the effect of computational tricks such as increasing the data with data augmentation, versus collecting more images from clinical procedures).

## 5. Conclusions

In conclusion, the clinical application of deep learning tools for musculoskeletal structure segmentation should be considered in the light of the strategies implemented for their development, to correctly explain their outcomes and evaluate their implications in the clinical domain. The availability of deep learning modules, integrated in medical devices for the segmentation of musculoskeletal structures could hasten and refine the precision of the treatment with the evolution of tailored devices, thus reducing human error and improving patient quality of life, while offering a new perspective of interpretability in the medical imaging domain and moving towards new frontiers in medical care.

## Figures and Tables

**Table 1:** Musculoskeletal anatomical structures and related bioimaging techniques investigated with deep learning approaches for object segmentation.

Anatomical Structures	Medical Imaging	References
Lower Limb	MRI	[5,7,19,25,26,27,28,29,30,31,32,33,34,35,36,37,38,39,40,41,42,43,44,45,46]
	US	[3,47,48,49]
	CT	[4,50,51,52]
	X-ray	[53]
Upper Limb	MRI	[54,55,56,57]
	US	[58,59]
	X-ray	[17,60]
Trunk	MRI	[11,18,61,62,63,64,65,66,67]
	CT	[14,15,16,68,69,70,71,72,73,74,75,76,77,78,79,80,81,82,83]
	MRI, CT, X-ray	[84]
	X-ray	[12,85,86,87,88]
Head	MRI	[89]
	CT	[6,8,9,10,90,91,92,93,94,95,96]
	CT, MRI	[97]
	X-ray	[98]
Pelvis	CT	[13,20,99]
Whole body	US	[1,2,100,101]
	CT	[102,103,104,105]

**Table 2:** Computational solutions to manage small sample datasets.

Computational Solution	Type	References
Data Augmentation	Affine transformations	[9,13,20,27,29,31,32,37,39,40,41,42,43,50,51,52,53,54,55,57,58,63,64,66,67,70,72,79,80,81,82,84,85,86,87,88,90,91,93,96,97,98,99,101,103,104,107]
Transfer Learning	-	[8,31,38,39,44,45,48,57,82,88,96]

**Table 3:** The most implemented pre-processing techniques.

Computational Solution	References
Normalization/histogram equalization	[1,12,13,15,17,18,19,25,26,28,31,32,33,39,40,55,59,60,61,63,70,73,80,85,86,90,91,104]
Intensity-based/dimensional-based filtering	[3,6,12,17,28,54,70,86]

**Table 4:** The most common deep learning network architecture applied to musculoskeletal structures, matched with bioimaging techniques.

Network Architecture	Medical Imaging	Reference
U-Net	MRI	[5,7,11,19,25,27,31,32,33,34,35,36,37,38,39,40,41,42,44,46,55,57,64,65,89,107]
	US	[49,100,101]
	CT	[4,9,13,15,16,59,69,71,72,73,76,77,78,79,80,81,83,90,91,93,94,95,99,103,104,105]
	X-ray	[12,17,53,60,85,86,87,88,98]

**Table 5:** Loss functions most frequently used for the segmentation of musculoskeletal structures.

Loss Function	Reference
DICE function or related variants	[4,7,8,10,12,15,19,31,37,38,39,46,49,50,52,55,57,59,67,72,73,80,85,91,94,99,101]
Cross entropy or variants	[1,2,4,5,9,26,28,29,32,33,36,43,50,51,71,74,87,88,93,97,100,105,107]
A combination of DICE + cross entropy loss function	[25,35,40,48,82,90,103]

**Table 6:** Performance indicators most frequently used for the segmentation of musculoskeletal structures.

Performance Indicators	Reference
DSC	[3,4,5,6,7,8,9,10,11,12,13,14,15,18,19,20,25,27,28,29,31,32,33,34,35,36,37,38,39,40,41,42,43,44,45,46,47,48,49,50,51,52,53,54,55,56,57,58,59,60,62,63,67,68,69,70,71,73,76,77,78,79,80,82,83,84,86,87,88,89,90,91,92,93,94,96,97,98,99,100,101,103,104,105,107]
HD	[4,6,10,18,28,34,41,44,47,48,52,58,68,73,86,90,91,96]
IoU	[2,3,7,9,10,11,18,28,33,34,35,47,48,50,52,57,59,67,68,72,73,77,78,81,85,88,89,93,101,103]
SD	[4,18,26,29,32,34,37,40,41,44,46,51,73,79,90,91,104,107]

**Table 7:** The most common post-processing operations.

Aim	Computational Solution	Reference
Segmentation refinement	Morphological operations (erosion, dilation…)	[12,13,26,59,80,91,101]
	Dimensional Thresholding	[10,12,15,17,25,26,36,73,95,104,107]
